# Propionic acid production from corn stover hydrolysate by *Propionibacterium acidipropionici*

**DOI:** 10.1186/s13068-017-0884-z

**Published:** 2017-08-17

**Authors:** Xiaoqing Wang, Davinia Salvachúa, Violeta Sànchez i Nogué, William E. Michener, Adam D. Bratis, John R. Dorgan, Gregg T. Beckham

**Affiliations:** 1 National Bioenergy Center, National Renewable Energy Laboratory, Golden, CO 80401 USA; 2 Chemical and Biological Engineering Department, Colorado School of Mines, Golden, CO 80401 USA

**Keywords:** Lignocellulosic biomass, Fermentation, Biochemicals, Biorefinery, Organic acids

## Abstract

**Background:**

The production of value-added chemicals alongside biofuels from lignocellulosic hydrolysates is critical for developing economically viable biorefineries. Here, the production of propionic acid (PA), a potential building block for C3-based chemicals, from corn stover hydrolysate is investigated using the native PA-producing bacterium *Propionibacterium acidipropionici*.

**Results:**

A wide range of culture conditions and process parameters were examined and experimentally optimized to maximize titer, rate, and yield of PA. The effect of gas sparging during fermentation was first examined, and N_2_ was found to exhibit improved performance over CO_2_. Subsequently, the effects of different hydrolysate concentrations, nitrogen sources, and neutralization agents were investigated. One of the best combinations found during batch experiments used yeast extract (YE) as the primary nitrogen source and NH_4_OH for pH control. This combination enabled PA titers of 30.8 g/L with a productivity of 0.40 g/L h from 76.8 g/L biomass sugars, while successfully minimizing lactic acid production. Due to the economic significance of downstream separations, increasing titers using fed-batch fermentation was examined by changing both feeding media and strategy. Continuous feeding of hydrolysate was found to be superior to pulsed feeding and combined with high YE concentrations increased PA titers to 62.7 g/L and improved the simultaneous utilization of different biomass sugars. Additionally, applying high YE supplementation maintains the lactic acid concentration below 4 g/L for the duration of the fermentation. Finally, with the aim of increasing productivity, high cell density fed-batch fermentations were conducted. PA titers increased to 64.7 g/L with a productivity of 2.35 g/L h for the batch stage and 0.77 g/L h for the overall process.

**Conclusion:**

These results highlight the importance of media and fermentation strategy to improve PA production. Overall, this work demonstrates the feasibility of producing PA from corn stover hydrolysate.

## Background

Lignocellulosic biomass is a promising feedstock for the production of sustainable biofuels [1] and building block chemicals [2]. Indeed, manufacturing high-value biobased chemicals alongside biofuels can both reduce reliance on petroleum-based feedstocks and simultaneously de-risk the financial viability of the integrated biorefinery [3–7]. Propionic acid (PA), an aliphatic C3 carboxylic acid, is a potentially promising value-added chemical that can be produced from lignocellulosic sugars. PA is a preservative and chemical intermediate in the food, pharmaceutical, and herbicide industries, and can serve as a potential building block for the production of various C3-based chemicals. Presently, PA is predominantly produced from fossil-based sources at industrial scales. However, recent efforts for PA production have focused on biological production using bacteria in the *Propionibacterium* genus, including *P. acidipropionici*, *P. freudenreichii*, and *P. shermanii*, which are able to metabolize a broad diversity of carbon sources and produce PA anaerobically. To date, multiple feedstocks have been explored in PA fermentation, including whey permeate [8–10], corn steep liquor (CSL) [11, 12], hydrolyzed corn meal [12, 13], glycerol [14–16], and Jerusalem artichoke tuber hydrolysate [17]. For PA production from lignocellulosic biomass, sugarcane bagasse hydrolysate [18, 19], enzymatically hydrolyzed aspen [20], and corncob molasses [21] have been used as substrates. In North America and elsewhere, corn stover is abundant and its utilization as a feedstock for PA production warrants investigation.

It is well known that PA is produced through the dicarboxylic acid pathway in *Propionibacteria* under anaerobic conditions [22, 23]. Unfortunately, this group of microorganisms simultaneously converts sugars to other carboxylic acids leading to a decreased PA yield. Succinic acid (SA) and acetic acid (AA) are the major byproducts [12–14, 16, 17, 24–26]. AA production from glucose in *Propionibacteria* is associated with redox balance, energy generation, and cell growth [22, 27]. Therefore, AA production is closely tied to substrate utilization rather than the process conditions. Lactic acid (LA) has also been previously demonstrated as an intermediate during PA fermentation [23, 28]. LA can be then converted to PA under glucose-limiting conditions by extending the fermentation time after glucose depletion. In addition, Stowers et al. demonstrated that 150 kPa of headspace pressure in the fermentor can maintain the LA titer under 3 g/L in batch fermentation [28].

Due to the strong product inhibition of acids, PA fermentations traditionally result in low volumetric productivity and titer [22]. Thus, some efforts have focused on in situ PA removal from the culture broth through extractive fermentation [11, 24, 29, 30], or development of high cell density (HCD) fermentation by means of cell immobilization [10, 12–14, 17, 19, 25] or through cell recycling via an external ultrafiltration system [9, 18]. Although these processes have been demonstrated as efficient approaches to alleviate inhibition and enhance PA productivity, the process complexity of controlling these bioreactors may ultimately limit their large-scale application [26]. On the other hand, densifying the concentration of the cell inoculum to conduct HCD fermentation is a simple and efficient way to reach high product titer and productivity. Recently, by increasing cell density, Stowers et al. reported a PA productivity to 2 g/L h from glucose [28]. Additionally, a PA productivity of 1.42 g/L h was reached from 50 g/L glycerol in HCD sequential batches [15]. Wang et al. also demonstrated a PA titer over 55 g/L with a productivity of 2.23 g/L h from pure glucose by conducting fed-batch HCD fermentation [26].

The aim of this work was to develop an efficient bioprocess for PA production using hydrolysate from lignocellulosic biomass—specifically from corn stover. The hydrolysate is produced via deacetylation, dilute acid pretreatment, and enzymatic hydrolysis (DDAPH) of corn stover, and includes sugars from both cellulose and hemicellulose fractions alongside various microbial inhibitors [e.g., acetate, furfural, and 5-hydroxymethylfurfural (HMF)]. PA production was first evaluated in mixed pure sugar streams containing inhibitors while N_2_ or CO_2_ was used to maintain an anaerobic environment. The feasibility of producing PA in DDAPH was subsequently examined in batch cultures, and the influence of different nitrogen sources and pH control reagents as well as byproduct accumulation were investigated. To increase PA titer, fed-batch fermentations using concentrated DDAPH in feed media were conducted and LA accumulation was also examined at different levels of nutrient supplementation. Finally, a fed-batch HCD fermentation mode was applied to further improve fermentation performance. This study demonstrates that an efficient, high-productivity, and high-titer process for PA production via fermentation of lignocellulosic hydrolysate is feasible.

## Results and discussion

### Evaluation of *P. acidipropionici* in mock DDAPH substrate and effects of CO_2_ and N_2_ on PA production

Dilute acid and hydrothermal pretreatment processes, such as the one utilized in the current study, often result in the formation of toxic compounds such as HMF, furfural, and acetic acid, and these compounds can inhibit microbial growth [31]. To evaluate the bacterial tolerance to such compounds, PA fermentation was first evaluated in mock DDAPH, which mimics the DDAPH composition, at an initial sugar concentration of ~60 g/L. Component concentrations in the mock DDAPH are provided in Table 1. Additionally, as *Propionibacterium* produces PA anaerobically, the effect of sparging N_2_ or CO_2_ on PA production during the fermentation was also analyzed. *P. acidipropionici* has been reported to be able to fix CO_2_ in the conversion step of pyruvate to oxaloacetate through pyruvate carboxylate [32, 33], which may allow for CO_2_ uptake and a corresponding PA yield enhancement during fermentation.

**Table 1:** Composition of DDAPH at the different dilution folds and mock DDAPH utilized in the current study

Compounds (g/L)	100% DDAPH	84% DDAPH	70% DDAPH	50% DDAPH	40% DDAPH	30% DDAPH	Mock DDAPH
Total sugars	153.6	129	107.6	76.8	61.4	46.3	58.9
Glucose	89.1	74.8	62.4	44.5	35.6	27.0	37.0
Xylose	56.7	47.6	39.7	28.3	22.7	17.0	19.0
Arabinose	7.8	6.6	5.5	3.9	3.1	2.3	2.9
Inhibitors							
Acetic acid	1.8	1.5	1.3	0.9	0.7	0.5	0.81
Furfural	0.9	0.8	0.6	0.4	0.4	0.3	0.34
HMF	0.14	0.12	0.1	0.07	0.06	0.04	0.05

**Fig. 1:**
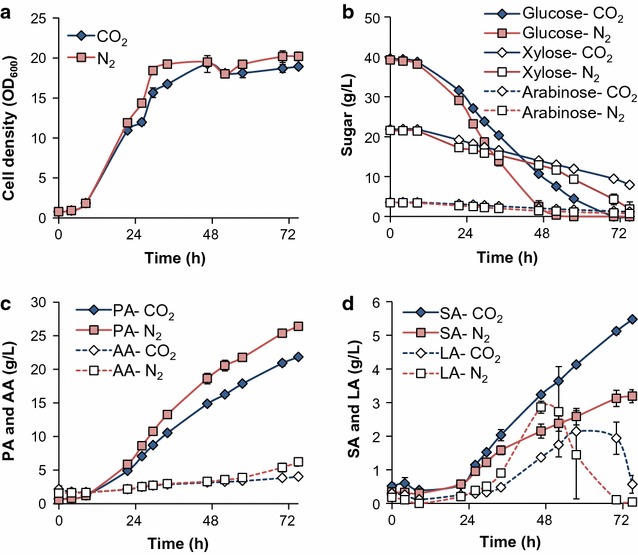
Batch fermentation kinetics of *P. acidipropionici* under CO_2_-sparging and N_2_-sparging conditions in Mock DDAPH. Scatter plots show **a** cell growth (OD_600_), **b** sugar utilization, **c** propionic and acetic acid production, and **d** succinic and lactic acid production. PA, propionic acid; AA, acetic acid; SA, succinic acid; LA, lactic acid

**Fig. 2:**
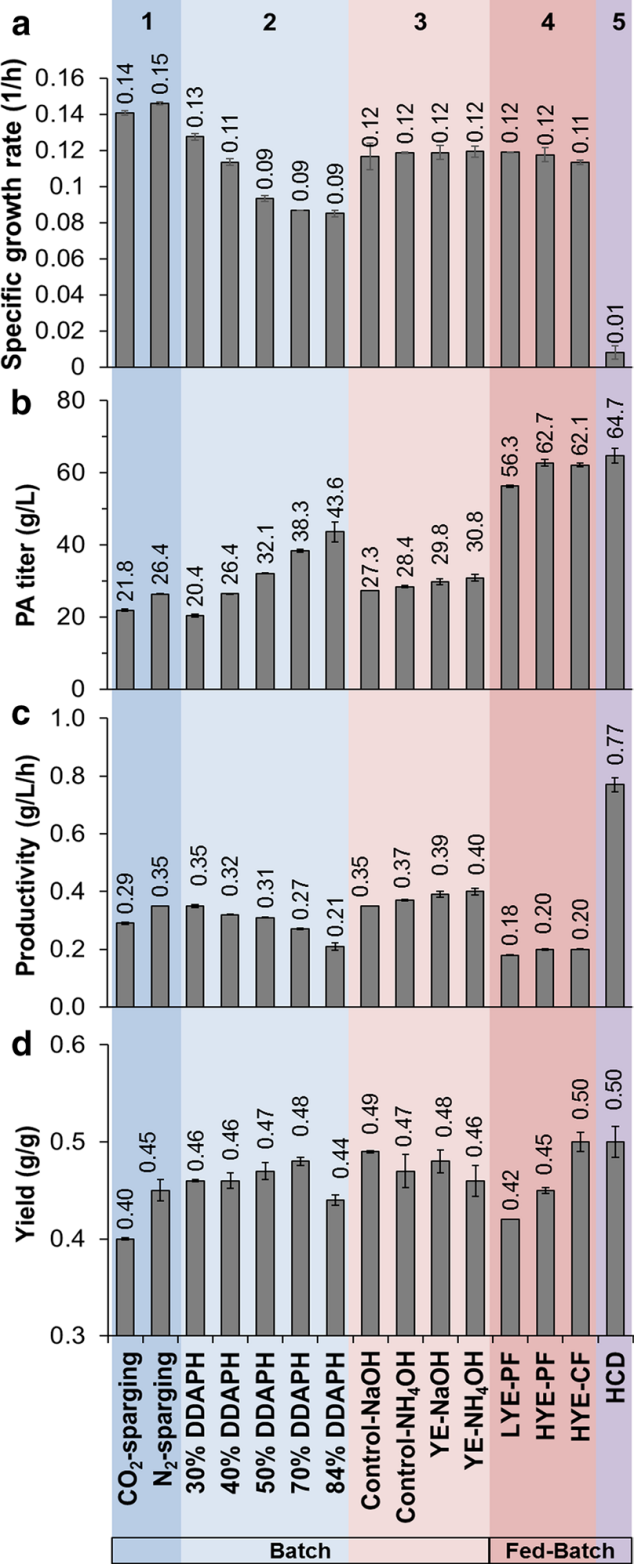
Kinetics of propionic acid fermentations by *P. acidipropionici* from the different experiments conducted in the current study. **a** Specific growth rate (1/h), **b** PA maximum titers (g/L), **c** overall productivities (g/L h), and **d** yields (g PA/g sugars) from all of the fermentations conducted in this work. Color blocks highlight different experiments: (1) effect of CO_2_ and N_2_ on PA production in mock DDAPH substrates, (2) effect of different initial DDAPH concentrations on PA production, (3) effect of different nitrogen sources and pH control reagents on PA production, (4) evaluation of PA production in different fed-batch modes and feeding media, and (5) fed-batch fermentation with high cell density. YE, yeast extract; Control, YE + TSB; LYE-PF, 13.9 g/L YE in feeding medium and pulsed feeding; HYE-PF, 86.8 g/L YE in feeding medium and pulsed feeding; HYE-CF, 86.8 g/L YE in feeding medium and continuous feeding; HCD, high cell density

Figure 1 shows the fermentation performance in mock DDAPH with both CO_2_ and N_2_-sparging. Sparging CO_2_ or N_2_ does not significantly affect cell growth (Figs. 1a, 2a), and sparging CO_2_ decreased sugar utilization rates (Fig. 1b). Figure 1b also shows that glucose, xylose, and arabinose can be used simultaneously by *P. acidipropionici*, with a higher glucose utilization rate than for the other sugars. As shown in Fig. 1c (and Fig. 2b), 26.4 g/L PA and 6.2 g/L AA were achieved at 75 h with N_2_, compared to the 21.8 g/L PA and 5.5 g/L AA obtained under CO_2_ sparging. The overall PA volumetric productivity (0.35 g/L h) and PA yields from sugars (0.45 g/g) under N_2_ were 20.7 and 12.5% higher than those obtained under CO_2_, respectively (Fig. 2c, d). Although the ability of *P. acidipropionici* ATCC 4875 to fix CO_2_ has been reported [32], the effect of CO_2_ assimilation on PA production was demonstrated to be highly dependent on the carbon source used in the fermentation [33]. Namely, Zhang et al. reported that CO_2_ exhibited minimal effect on PA production from glucose, but significantly enhanced cell growth and PA and SA production when using glycerol as a sole carbon source [33]. In the current study, we observed reduced sugar utilization rates, lower PA titers, and lower PA productivity and yield from mixed sugars under CO_2_-sparging than with N_2_.

*Propionibacteria* natively produce other products, such AA, SA, and LA (vide supra). Since the AA production pathway can generate energy in the form of ATP and, together with PA production, achieve redox balance [22], it is expected and observed that AA is produced along with PA (Fig. 1c). In parallel, LA accumulation was observed during glucose utilization (Fig. 1d). However, when glucose was depleted, the LA concentration decreases to zero. Under N_2_-sparging, the maximum LA concentration was 2.9 g/L (at 46.5 h) when the glucose concentration was 2.4 g/L. Consistent with the slower glucose consumption rate under CO_2_-sparging, the maximum LA concentration (2.1 g/L) peaked later (57.5 h). Unlike the other acids, SA accumulation exhibited the opposite trend under the two conditions. After 26 h, SA production rates were enhanced with CO_2_, and the final SA concentration was 71% higher with CO_2_ than N_2_. This type of increase in SA production when utilizing CO_2_ or CO_2_ donors has been previously demonstrated in other capnophilic and SA-producing organisms [34]. Overall, these results demonstrate that (i) *P. acidipropionici* tolerates some of the known primary inhibitors in corn stover hydrolysate and that (ii) using N_2_ to maintain anaerobic conditions is advantageous for selective PA production. Thus, N_2_ sparging was applied in the subsequent experiments.

### Effect of different initial DDAPH concentrations on *P. acidipropionici*

**Fig. 3:**
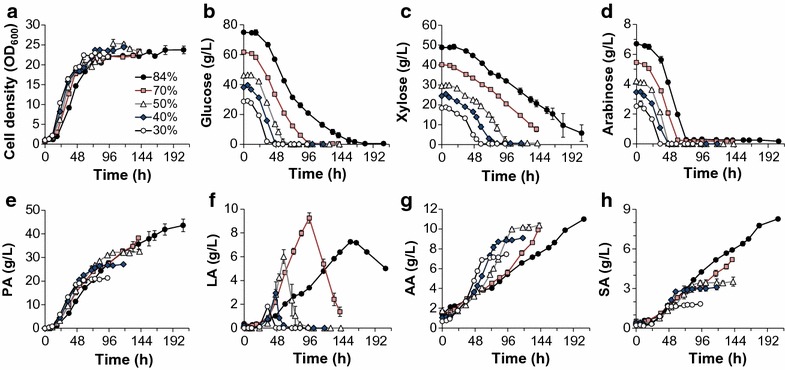
Batch fermentation profiles of *P. acidipropionici* in different DDAPH concentrations. Scatter plots show **a** cell growth (OD_600_), **b** glucose utilization, **c** xylose utilization, **d** arabinose utilization, **e** PA production, **f** LA production, **g** AA production, and **h** SA production

PA production was subsequently evaluated utilizing different DDAPH concentrations from 30 to 84% (Table 1). The resulting fermentation data are shown in Fig. 3 and fermentation parameters are summarized in Fig. 2. The effect of minor lignocellulosic inhibitors (apart from furfural, HMF, and acetic acid) on the growth of *P. acidipropionici* can be observed by comparing the specific growth rates when cultivated on mock hydrolysate (Table 1), and diluted DDAPH (in this case 40%, to have the same initial concentration) (Fig. 2a). A reduction of 26.7% (from 0.15 1/h to 0.11 1/h) was observed, demonstrating the contribution of additional compounds, such as lignin-derived aromatics [4, 31], to the inhibitory level of lignocellulosic hydrolysate. While an expected decrease of specific growth rate occurred when increasing DDAPH content (Figs. 2a, 3a) due to both increasing inhibitors and sugar concentrations, a similar growth rate (0.09 1/h) was obtained from 50 to 84% DDAPH (Fig. 2a), highlighting the tolerance of *P. acidipropionici* towards hydrolysate. Rather than initial DDAPH concentration, a more severe inhibition on cell growth was observed from PA accumulation. At ~70 h, when the PA concentration reached >20 g/L, a concentration previously reported as inhibitory [35], cell growth ceased and entered a stationary phase (Fig. 3a). As shown in Fig. 3b–d, the consumption rates of each sugar were similar from 30 to 50% DDAPH, whereas decreased glucose and xylose utilization rates were observed at 70 and 84% DDAPH dilutions. Moreover, utilization of xylose was incomplete at the two highest DDAPH concentrations after 140 h of incubation.

PA production from different DDAPH media exhibited some noteworthy differences (Fig. 3e). After an 11-h lag, the PA concentrations increase almost linearly along with the exponential cell propagation. PA production slows and ceases when the sugars were completely consumed in 30–50% DDAPH. Conversely, the PA titer continues to increase in 70 and 84% DDAPH even though cells reach the stationary phase after ~70 h. The highest PA titer of 43 g/L was obtained at 207.5 h in 84% DDAPH. Overall, the PA production rate decreases with increasing DDAPH content. The highest PA volumetric productivity (0.35 g/L h) was achieved from 30% DDAPH medium, and the lowest (0.21 g/L h) at 84% DDAPH (Fig. 2c). PA yield ranged from 0.44 to 0.48 g/g (Fig. 2d), demonstrating that the hydrolysate concentration is not as critical for PA yield as for productivity.

In terms of other byproducts, the AA production rate was higher at lower DDAPH concentrations, which is consistent with the cell growth and PA production rates (Fig. 3g). Significant amounts of LA and SA were also formed, especially with more concentrated DDAPH (Fig. 3f, h). However, LA was consumed after glucose was depleted.

For further fermentations, although 30% DDAPH gave the highest PA productivity, the PA titer was deemed too low for economically viable downstream separations. Since 50% DDAPH led to the same PA productivity and a higher PA titer relative to 40% DDAPH, and presented the highest productivity and complete utilization of sugars when compared to less diluted media, 50% diluted DDAPH was employed for subsequent fermentations.

### Effect of different nitrogen sources and pH control reagents on *P. acidipropionici*

**Fig. 4:**
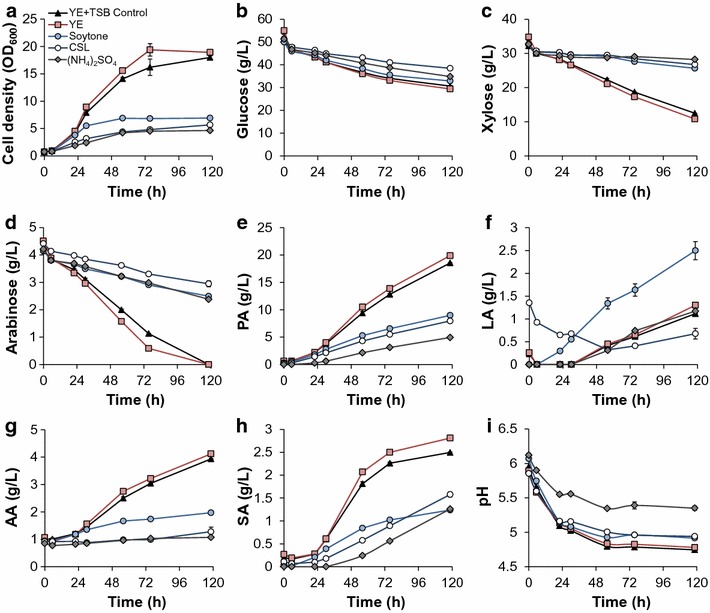
Time course fermentation profiles of *P. acidipropionici* under different nitrogen sources in serum bottles. *Scatter plots* show **a** cell growth (OD_600_), **b** glucose utilization, **c** xylose utilization, **d** arabinose utilization, **e** PA production, **f** LA production, **g** AA production, **h** SA production, **i** pH. The nitrogen source in the control case contains both YE and TSB

In addition to the carbon source, efficient PA fermentation by *Propionibacterium* requires nitrogen-containing media. Therefore, with 50% DDAPH hydrolysate, several nitrogen sources including an inorganic salt ((NH_4_)_2_SO_4_), processed yeast products (yeast extract, YE), a less expensive soy product (soytone), and a byproduct of the corn wet milling process (corn steep liquor, CSL) were screened in serum bottles. The serum bottles contained the same total *N* concentration (measured in g/L) and the combination of 10 g/L YE and 5 g/L TSB was used as a control. The results show that YE and the control media produce the two highest cell densities with YE medium being marginally better (Fig. 4a). In contrast, soytone, (NH_4_)_2_SO_4_, and CSL produce limited cell biomass. The same trend was observed in sugar utilization rates, in particular for xylose and arabinose (Fig. 4c, d). Figure 4c shows that over 61% xylose was consumed in YE and the control medium, while only a small amount of xylose was consumed in the other media. Moreover, arabinose was completely utilized in YE and the control media after 118.5 h, compared to the ~60% arabinose remaining in the other media (Fig. 4d). As a result, the highest acid titers were observed in YE and the control media except for LA (Fig. 4e–h). Unlike the other acids, the highest accumulation of LA was reached in soytone (Fig. 4f).

Generally, the lack of multiple nutrients, such as vitamins and/or various amino acids, can lead to poor fermentation performance, and this could be the reason for the low bacterial growth on inorganic nitrogen; however, bacterial performance in CSL media was similar. Although some references report PA production from CSL, as both carbon and nitrogen substrates [11, 12], CSL only exhibits slight advantages over (NH_4_)_2_SO_4_. Additionally, considering the high LA generation and lower PA production in soytone, this product was not further employed as a nitrogen source. As a result, YE and the control conditions (YE + TSB) were utilized as nitrogen sources going forward.

**Fig. 5:**
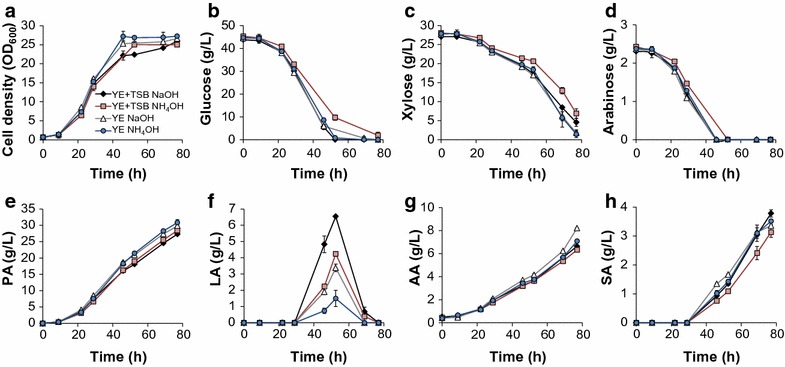
Batch fermentation profiles of *P. acidipropionici* under different nitrogen sources and pH control methods. Control: TSB + YE. Scatter plots show **a** cell growth (OD_600_), **b** glucose utilization, **c** xylose utilization, **d** arabinose utilization, **e** PA production, **f** LA production, **g** AA production, and **h** SA production

Given the lack of pH control in the serum bottle experiments, YE and control nitrogen sources were further evaluated in bioreactors at a controlled pH (6.0). NaOH [9, 12, 13, 16, 17, 19, 21, 24, 29, 33] and NH_4_OH [8, 14, 15, 26, 28] are the most commonly used bases during PA fermentation; however, to our knowledge, no comparison of these two bases has been reported previously. As such, we investigated the effects of 4 M NaOH and 4 M NH_4_OH on PA production in both YE and the control media. The combination of the control medium and 4 M NaOH also serves as control here, as these conditions were used in previous fermentations. The detailed fermentation data are shown in Fig. 5 and summarized in Fig. 2.

The fermentation profiles are similar under the different conditions before 29 h. Subsequently, differences became more obvious, particularly in LA production (Fig. 5f). LA accumulation occurs after 29 h and peaks at 52.5 h in all cultures. LA exhibits the highest concentrations (~7 g/L) in the control (TSB + YE) using both bases to adjust the pH. In contrast, the lowest LA accumulation (~1.5 g/L) occurs when using YE and NH_4_OH.

It is worth noting that, at approximately 80 h, LA decreases to zero in all cases and thus, the yields are similar (Fig. 2). This finding is relevant for fed-batch fermentations since, if sugars are not maintained at low levels, LA will likely accumulate. Stowers et al. pressurized the reactor headspace at 150 kPa by using N_2_ to prevent the LA titer from exceeding 3 g/L in a batch process, with the hypothesis that higher pressure increased the soluble CO_2_ concentration in the culture, in turn enhancing CO_2_ fixation by pyruvate carboxylase and converting pyruvate to PA instead of LA [28]. Although our results indicate that CO_2_-sparging indeed decreases the maximum LA concentration during batch fermentation compared to N_2_-sparging, the difference is less than 1 g/L (Fig. 1d). Moreover, CO_2_-sparging exhibits adverse effects on PA production (Fig. 1c). Therefore, we instead decrease LA production via use of YE as a sole nitrogen source and by replacing NaOH with NH_4_OH for pH control, which results in the lowest observed LA accumulation. Finally, the maximum PA titer (30.8 g/L) and productivity (0.40 g/L h) were obtained when using YE and NH_4_OH, which were 13 and 14% higher than the control results, respectively (Fig. 2).

### Evaluation of PA production by *P. acidipropionici* in different feeding modes and media

The PA volumetric productivity was enhanced over 14% just by optimizing nitrogen sources and pH control reagents in batch mode (vida supra). However, high PA titer, which is critical for cost-effective recovery and purification downstream, is limited by the initial sugar concentration in batch operation. Therefore, fed-batch fermentation could increase PA titers and alleviate substrate inhibition.

**Fig. 6:**
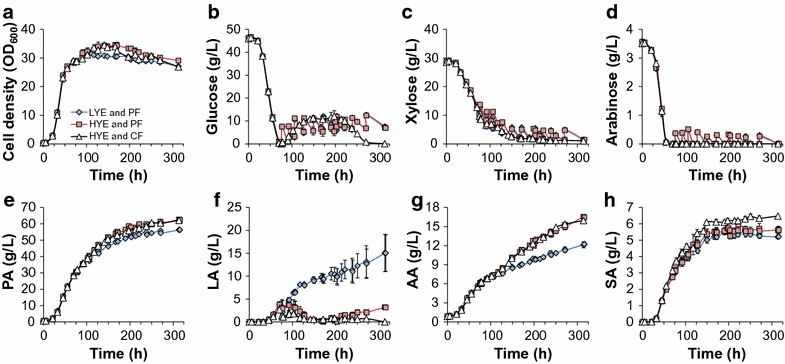
Fed-batch fermentation profiles of *P. acidipropionici* while utilizing DDAPH and different YE concentrations. LYE, low yeast extract concentration (13.9 g/L); HYE, high yeast extract concentration (86.8 g/L) and different feed controls (PF, pulsed feeding; CF, continuous feeding). Scatter plots show **a** cell growth (OD600), **b** glucose utilization, **c** xylose utilization, **d** arabinose utilization, **e** PA production, **f** LA production, **g** AA production, and **h** SA production

One strategy to improve fed-batch fermentation performance is to optimize the nutrient profile of the feed medium. Concentrated nitrogen and sugar sources have been previously utilized as a feed for PA production in fed-batch fermentations [11, 12], whereas other studies only feed a concentrated carbon source [13, 17, 19, 24]. To our knowledge, no previous studies analyzed the influence of nitrogen concentration in the feed medium on PA production in fed-batch fermentation. Another strategy to improve fed-batch fermentation relies on the pattern of feed addition. The most common feeding strategy applied in PA fermentation is pulsed feeding (PF) [11–13, 17, 19, 21, 24–26]. A more common approach for feeding control is continuous feeding (CF), wherein the carbon source is fed at similar rates as its utilization. To determine if the feed composition and/or feeding strategy could improve PA production, PF with concentrated DDAPH (500 g/L total sugars) and 13.9 g/L (low YE, or LYE) or 86.8 g/L YE (high YE, or HYE), and CF with concentrated DDAPH (500 g/L total sugar content) and 86.8 g/L YE (HYE) were compared. The fermentation data are shown in Fig. 6 and fermentation performance parameters are summarized in Fig. 2.

In the three cases, 50% DDAPH was used in the batch phase and the feeding started at 77 h when glucose was depleted. For PF, the cultures with a feed medium containing LYE and HYE media exhibit nearly identical sugar utilization profiles (Fig. 6b–d), indicating that differences in YE concentration during feeding do not affect sugar utilization. Additionally, an interesting phenomenon regarding sugar utilization was observed. Despite that the xylose consumption rate (0.25 g/L h) was much lower than the glucose consumption rate (0.63 g/L h) in the batch phase, no xylose accumulation occurred during the feeding phase. In fact, xylose consumption rates were comparable to those obtained with glucose in the three cases (Fig. 6c). It is well known that sequential utilization of different sugars is caused by carbon catabolite repression (CCR), which is generally related to a multiprotein phosphorelay system called the phosphotransferase system (PTS) [36]. Although various sugars can be utilized by *P. acidipropionici* simultaneously, glucose is preferred to xylose (vida supra). Parizzi et al. suggested the presence of a CCR system by glucose in *P. acidipropionici* by identifying 23 genes in its genome, and also by comparing its growth rates in various carbon sources with and without hexose analogous 2-deoxy-glucose [32]. Moreover, it has been previously reported in *Saccharomyces cerevisiae* [37, 38] and *Enterococcus mundtii* [39] that glucose catabolite repression can be avoided by maintaining a low concentration of glucose. In this study, CCR was avoided by maintain the glucose concentration below 15 g/L, and as a consequence an enhanced xylose uptake rate by *P. acidipropionici* was observed during the feeding phase.

Although no differences in sugar utilization rates were observed between LYE and HYE media, HYE slightly enhanced the cell growth and PA production in both PF and CF. Unlike in batch cultures, in which the OD_600_ plateaued when the PA concentration was >20 g/L, the cell density continued increasing in all the fermentations after 55.5 h (when the PA concentration exceeded 21 g/L). However, cells in LYE medium reached the stationary phase earlier than cells in HYE media (91 and 167 h, respectively) and exhibit lower maximum OD_600_ (30.8 and 34, respectively) likely due to nutrient limitations (Fig. 6a).

HYE media also led to an increase of acids production, except for LA. At the end of fermentation, 62.1 and 62.7 g/L PA were achieved from HYE medium with PF and CF, respectively. These titers were 11% higher than that obtained in LYE medium (56.3 g/L) (Figs. 6e, 2b). Similar to PA production, both cultures grown using the HYE medium produced higher acid concentrations relative to LYE, specifically 34% for AA and 25 and 8.1% for SA (PF and CF, respectively) (Fig. 6g, h). Oppositely, LA concentrations in HYE medium were maintained below 4 g/L over the whole process (Fig. 6f), in contrast to 15 g/L titer in LYE medium. In all fermentations, LA production begun at 45.5 h and its consumption occurred after 70 h when glucose was depleted in the batch phase. At 91, 14 h after the beginning of the feeding phase, LA accumulation was observed again because of glucose feeding. However, during the following 80 h, the LA concentration in HYE medium neared zero in the presence of glucose, contrary to the increased LA accumulation in LYE medium. Moreover, by comparing LA concentrations between HYE CF and PF before 250 h, when glucose concentrations were above 4 g/L in both cultures, CF leads to a lower LA production.

Yang et al. evaluated different levels of organic nitrogen supplements (yeast extract and trypticase) on PA production from whey permeate in continuous cell-immobilized fermentation, and found that cell density, PA, and AA levels were higher when increasing nutrient levels, whereas SA exhibited the opposite trend [10]. It was also suggested that for long-term continuous fermentation, nutrient limitation could limit cell growth while maintaining cell activity and direct more carbon to target product [10]. In the current study instead, SA production was enhanced along with PA and AA in HYE medium. LA accumulation was significantly higher in LYE medium; however, this acid was not evaluated by Yang and co-workers.

Overall, by increasing the YE concentration in the feed medium and applying a CF strategy, PA titer, productivity, and yield were increased by 10, 11, and 14% compared to those from LYE PF culture, respectively (Fig. 2). Thus, HYE concentrations and CF mode were selected for the final experiments.

### Fed-batch fermentation with high cell density (HCD)

**Fig. 7:**
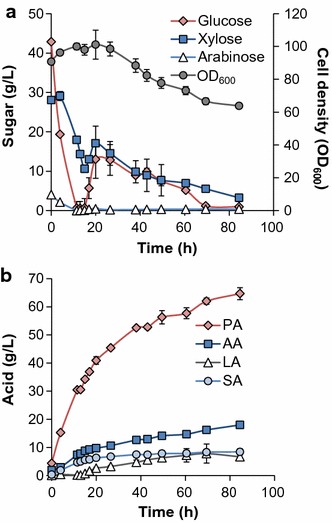
High cell density (HCD)—fed-batch fermentation profiles. Scatter plots show **a** cell growth (OD_600_) and sugar utilization and **b** acid production

Although high PA titer and limited LA accumulation were achieved via fed-batch cultivation, PA productivity was only 50% of that obtained in batch fermentation. With the purpose of increasing both titer and productivity simultaneously, fed-batch HCD fermentations were conducted. The detailed fermentation profiles are shown in Fig. 7 and results are summarized in Fig. 2. Bioreactors were inoculated at an initial OD_600_ of 91 (corresponding to a 23.5 g CDW/L). No lag was observed and the OD_600_ increases to over 100 at 20 h in the batch phase (Fig. 7a). Sugars were consumed in less than 20 h and, in the case of glucose and arabinose, complete consumption was observed within 11.5 h (Fig. 7a). When the feeding was initiated (at 15 h), the PA concentration was 30.5 g/L and the PA productivity was 2.35 g/L h (Fig. 2). The OD_600_ declines to 64 in the following 64.5 h, and the PA titer increases monotonically to 64.7 g/L at 84.5 h with an overall productivity of 0.77 g/L h (Fig. 2).

Additionally, compared to the previous fed-batch fermentation in HYE CF culture, a slight increase of other acids was observed in fed-batch HCD fermentation; however, PA yields were similar in both cases (0.5 g/g) (Fig. 2), likely due to the reduction of cell biomass production. Overall, the HCD fed-batch fermentation improves PA productivity 2.8-fold compared to the fed-batch process while reaching the same titer and yield (Fig. 2). Compared to the batch fermentation, fed-batch HCD fermentation improved PA titer and productivity by 110% and 92.5% (Fig. 2), respectively.

Three previous reports have employed *Propionibacterium* to produce PA from lignocellulose hydrolysate and are of interest for comparative purposes. Ramsay et al. reported a PA titer of 22.9 g/L in batch cultures from aspen with a productivity of 0.30 g/L h [20]; Liu et al. used corncob molasses to obtain a PA titer of 71.8 g/L and a productivity of 0.28 g/L h in a fed-batch fermentation [21], and Zhu et al. reported a PA concentration of 58.8 g/L at a productivity of 0.38 g/L h from cell-immobilized fed-batch fermentation in sugarcane bagasse hydrolysate [19]. Taken together, our results are among the highest PA productivity and titer reported to date from lignocellulosic feedstocks.

### Conclusions

Here, we report anaerobic PA production by *P. acidipropionici* from corn stover, an abundant, industrially relevant, renewable lignocellulosic resource. Through a systematic investigation of different fermentation conditions and strategies, the PA titer and productivity were improved from 32.1 g/L and 0.31 g/L h from DDAPH batch fermentation to 64.7 g/L and 0.77 g/L h from DDAPH fed-batch HCD fermentation, respectively, roughly doubling both the titer and the productivity through changes to process conditions. Moreover, this work demonstrated the influence of nitrogen source and its concentration on LA accumulation, a major byproduct that has not been previously addressed in most of the publications describing PA fermentation.

Going forward, both the strain and the fermentation process could be further improved for industrial viability and integration into a lignocellulosic biorefinery. For instance, engineered *P. acidipropionici* with high acid tolerance [40] could be evaluated in lignocellulosic hydrolysates to enhance both PA titer and productivity. In addition, to reduce downstream process cost and increase yields, the metabolic pathway of LA production could be knocked out and industrial medium components could be used to replace the more costly laboratory-grade counterparts.

## Methods

### Hydrolysate preparation

Pilot-scale production of DDAPH was conducted as described by Shekiro et al. [41] with some noted modifications. Corn stover, provided by Idaho National Laboratory, was hammer-milled and filtered with a rejection screen. Milled fibers were then deacetylated using a dilute NaOH solution (0.4% w/w) at 80 °C for 2 h. Following deacetylation, remaining solids were rinsed with water and then mixed with dilute H_2_SO_4_ solution to achieve a 0.8% (w/w) acid concentration for dilute acid pretreatment. The slurry was thoroughly mixed for 2 h at room temperature, dewatered to approximately 40% solids, and then incubated in a horizontal pretreatment reactor at 160 °C with a residence time of 10 min. After pretreatment, the material was separated into the slurry stream with high solid content and volatile flash vent stream. Pretreated deacetylated dry slurry was neutralized using a 50% NaOH solution, and diluted with the addition of process water to 20% total solids. Novozymes Cellic^®^ CTec2 was added to the slurry, and the mixture was incubated in a 130-L Jaygo paddle reactor at ~50 °C for 7 days under constant agitation. The sugar stream separated from lignin solids was then pH-adjusted to 6 and used as carbon substrate for PA fermentation.

### Microorganism and media

Native *P. acidipropionici* ATCC 4875 was purchased from the American Type Culture Collection. The bacterium was initially revived in serum bottles under an anaerobic environment in the media detailed below. When the culture reached the exponential phase, it was preserved in 15% (v/v) glycerol stock and stored at −80 °C.

The bacterial medium contained 10 g/L yeast extract (YE) (BD Bacto™), 5 g/L tryptic soy broth (TSB) (BD™), 0.48 g/L K_2_HPO_4_ (Sigma-Aldrich), 0.98 g/L KH_2_PO_4_ (Sigma-Aldrich), and 0.05 g/L MnSO_4_ (Sigma-Aldrich) with varying amounts and types of carbon sources (glucose, sugars from mock DDAPH, and sugars from DDAPH). Media supplemented with 40 g/L glucose were used for cell revival and the seed culture. Mock DDAPH, which is the first medium evaluated in the current study, mimics the composition of DDAPH. Mock DDAPH contained glucose, xylose, and arabinose, as major sugar components in DDAPH, at a total sugar concentration of 60 g/L but also well-known inhibitors found in DDAPH, such as furfural, HMF, and AA. The percentages of each compound in the mock DDAPH were based on the actual composition of DDAPH at 40% dilution (Table 1). Then, DDAPH at different concentrations was evaluated for PA fermentation. The sugar and inhibitor concentrations in different diluted DDAPH streams are also listed in Table 1. For fed-batch fermentations, DDAPH concentrated using a rotary evaporator (Hei-VAP value, Heidolph) was used as feed to a concentration of 500 g/L total sugars.

To optimize the nitrogen source, YE (5 g/L) and TSB (10 g/L) were directly replaced with different types of inorganic and organic nitrogen sources, including 7.1 g/L (NH_4_)_2_SO_4_ (Sigma-Aldrich), 13.9 g/L yeast extract, 16.1 g/L soytone (BD Bacto™), and 16.4 g/L CSL (Sigma-Aldrich). The concentration of each nitrogen source was calculated based on its total N content reported by manufacturers to ensure that all media shared the same total N concentration (g/L). Mock-DDAPH and DDAPH substrates were sterilized using a Nalgene vacuum filtration system with a pore size of 0.2 µm. Nitrogen sources and salt components were autoclaved separately and aseptically combined to the target concentration.

### Fermentation conditions for propionic acid production

Seed cultures, revived from glycerol stocks, were incubated in serum bottles at 30 °C for 48 h and then used to inoculate (7%, v/v) the fermentation medium. In order to wash metabolites and unconsumed sugars from the seed culture into the fermentation medium, cells were previously pelleted by centrifugation at 6000*g* for 10 min at 4 °C and then re-suspended in fresh medium to seed batch fermentations at an initial optical density at 600 nm (OD_600_) of approximately 0.6. All fermentations (excluding seed culture propagation for HCD fermentations) were conducted in 500-mL fermentation vessels (BIOSTAT Qplus, Sartorius) containing 300 mL medium with constant agitation at 300 rpm. The bioreactor temperature was maintained at 30 °C and the pH at 6.0 by the addition of 4 M NaOH (Sigma-Aldrich) or 4 M NH_4_OH (Sigma-Aldrich). Anaerobic conditions were established by sparging the medium with N_2_ or CO_2_ during the fermentation process at 0.2 vvm.

In fed-batch fermentation, when the initial total sugar concentration of ~80 g/L from 50% DDAPH was almost depleted, concentrated DDAPH containing 500 g/L total sugars and different concentrations of yeast extract were pulse-fed into the reactor. Continuous feeding was also performed by continuously pumping the concentrated hydrolysate into bioreactors. Sugar content was analyzed frequently and the pumping speed was adjusted to ensure that the sugar-feeding rate was equal to the sugar-consumption rate. Solution of 4 M NaOH or NH_4_OH was used to maintain the pH at 6.0 and anaerobic conditions were maintained through N_2_ sparging at 0.2 vvm.

For the fed-batch HCD fermentations, seed cultures were produced in 5-L bioreactors (New Brunswick) containing 3 L of bacterial medium supplemented with glucose as carbon source (40 g/L). Cultivations were performed under the same conditions as detailed before until culture reached mid to late exponential phase with OD_600_ of ~26. Cell pellets from 1.05 L seed culture were harvested by centrifugation and then resuspended in fresh DDAPH medium to seed 300 mL HCD fermentation medium, giving the initial OD_600_ values of ~91. The carbon sources in the batch and feed media were same with those used in fed-batch fermentation, and the concentrations of YE and salts were doubled. Continuous feed was applied in fed-batch HCD fermentation as detailed above. N_2_ and 4 M NH_4_OH were applied as previously described.

All the fermentations were performed in duplicate. Results are given as the average of the duplicate and error bars with standard error across duplicate fermentations.

### Analytical methods

Cell growth was followed by measuring OD_600_ with a spectrophotometer. To eliminate color interferences from the dark DDAPH media, samples were centrifuged at 12,000 rpm for 5 min and then the cell-free supernatant was measured as the background. Dry cell weight (DCW) was determined as described by Wang et al. [26]. The cells were then collected by centrifugation, washed with distilled H_2_O, and dried until constant DCW. A linear relationship between the value of OD_600_ and DCW was established by plotting OD_600_ as a function of DCW with one unit of OD_600_ equal to 0.2584 g/L DCW. The concentrations of sugars (glucose, xylose, and arabinose), organic acids (PA, AA, SA, LA), furfural and HMF were analyzed by an Agilent 1200 (Agilent Technologies, Santa Clara, CA) series HPLC system. Analytes were separated utilizing a Bio-Rad (BioRad, Hercules, CA) Aminex^®^ HPX-87H, 9 μm × 300 mm × 7.8 mm Ion Exclusion Column (BioRad) at 55 °C with a mobile phase of 0.01 N H_2_SO_4_ at a flow rate of 0.6 mL/min. Individual standards were purchased from Sigma-Aldrich (Sigma-Aldrich). The levels of the calibration curve ranged from 0.05 to 50 mg/mL. A minimum of 5 calibration levels was used with an *r* ^2^ coefficient of 0.995 or better for each analyte. A check calibration standard was analyzed every 10 samples to insure the integrity of the initial calibration.

### Calculation of PA yield and productivity

PA yield from the consumed sugars was calculated as the total PA produced (g) divided by total sugar consumed (g) at the end of fermentation. The PA overall volumetric productivity (g/L h) was calculated as the PA concentration (g/L) divided by the fermentation time (h).

## Abbreviations

AA: acetic acid
CF: continuous feeding
CSL: corn steep liquor
DCW: dry cell weight
DDAPH: deacetylated, dilute acid pretreated and enzymatic hydrolyzed corn stover hydrolysate
HCD: high cell density
HMF: 5-hydroxymethylfurfural
HPLC: high-pressure liquid chromatography
HYE: high yeast extract concentration
LA: lactic acid
LYE: low yeast extract concentration
PA: propionic acid
PF: pulsed feeding
SA: succinic acid
TSB: tryptic soy broth
YE: yeast extract

## References

[1] Chundawat SP, Beckham GT, Himmel ME, Dale BE (2011). Deconstruction of lignocellulosic biomass to fuels and chemicals. Chem Biomol Eng.

[2] Gallezot P (2012). Conversion of biomass to selected chemical products. Chem Soc Rev.

[3] Bozell JJ, Petersen GR (2010). Technology development for the production of biobased products from biorefinery carbohydrates—The US Department of Energy’s “top 10” revisited. Green Chem.

[4] Salvachúa D, Mohagheghi A, Smith H, Bradfield MF, Nicol W, Black BA, Biddy MJ, Dowe N, Beckham GT (2016). Succinic acid production on xylose-enriched biorefinery streams by *Actinobacillus succinogenes* in batch fermentation. Biotechnol Biofuels.

[5] Salvachúa D, Smith H, St John PC, Mohagheghi A, Peterson DJ, Black BA, Dowe N, Beckham GT (2016). Succinic acid production from lignocellulosic hydrolysate by *Basfia succiniciproducens*. Bioresour Technol.

[6] Biddy MJ, Davis R, Humbird D, Tao L, Dowe N, Guarnieri MT, Linger JG, Karp EM, Salvachúa D, Vardon DR (2016). The techno-economic basis for coproduct manufacturing to enable hydrocarbon fuel production from lignocellulosic biomass. ACS Sustain Chem Eng..

[7] Davis R, Biddy MJ, Tan E, Tao L, Jones SB. Biological conversion of sugars to hydrocarbons technology pathway. Office of Scientific & Technical Information Technical Reports. 2013.

[8] Blanc P, Goma G (1987). Propionic acid fermentation: improvement of performances by coupling continuous fermentation and ultrafiltration. Bioprocess Eng.

[9] Boyaval P, Corre C (1987). Continuous fermentation of sweet whey permeate for propionic acid production in a CSTR with UF recycle. Biotech Lett.

[10] Yang ST, Zhu H, Li Y, Hong G (1994). Continuous propionate production from whey permeate using a novel fibrous bed bioreactor. Biotechnol Bioeng.

[11] Ozadali F, Glatz BA, Glatz CE (1996). Fed-batch fermentation with and without on-line extraction for propionic and acetic acid production by *Propionibacterium acidipropionici*. Appl Microbiol Biotechnol.

[12] Paik HD, Glatz BA (1994). Propionic acid production by immobilized cells of a propionate-tolerant strain of *Propionibacterium acidipropionici*. Appl Microbiol Biotechnol.

[13] Huang YL, Wu Z, Zhang L, Cheung CM, Yang ST (2002). Production of carboxylic acids from hydrolyzed corn meal by immobilized cell fermentation in a fibrous-bed bioreactor. Bioresour Technol.

[14] Dishisha T, Alvarez MT, Hatti-Kaul R (2012). Batch- and continuous propionic acid production from glycerol using free and immobilized cells of *Propionibacterium acidipropionici*. Bioresour Technol.

[15] Dishisha T, Stahl A, Lundmark S, Hatti-Kaul R (2013). An economical biorefinery process for propionic acid production from glycerol and potato juice using high cell density fermentation. Bioresour Technol.

[16] Wang Z, Yang ST (2013). Propionic acid production in glycerol/glucose co-fermentation by *Propionibacterium freudenreichii* subsp. *shermanii*. Bioresour Technol.

[17] Liang ZX, Li L, Li S, Cai YH, Yang ST, Wang JF (2012). Enhanced propionic acid production from Jerusalem artichoke hydrolysate by immobilized *Propionibacterium acidipropionici* in a fibrous-bed bioreactor. Bioprocess Biosyst Eng.

[18] Crespo JPSG, Moura MJ, Carrondo MJT (1990). Some engineering parameters for propionic acid fermentation coupled with ultrafiltration. Appl Biochem Biotechnol.

[19] Zhu L, Wei P, Cai J, Zhu X, Wang Z, Huang L, Xu Z (2012). Improving the productivity of propionic acid with FBB-immobilized cells of an adapted acid-tolerant *Propionibacterium acidipropionici*. Bioresour Technol.

[20] Ramsay JA, Aly Hassan MC, Ramsay BA (1998). Biological conversion of hemicellulose to propionic acid. Enzym Microb Technol.

[21] Liu Z, Ma C, Gao C, Xu P (2012). Efficient utilization of hemicellulose hydrolysate for propionic acid production using *Propionibacterium acidipropionici*. Bioresour Technol.

[22] Wang Z, Sun J, Zhang A, Yang ST (2013). Propionic acid fermentation.

[23] Choi CH, Mathews AP (1994). Fermentation metabolism and kinetics in the production of organic acids by *Propionibacterium acidipropionici*. Appl Biochem Biotechnol.

[24] Jin Z, Yang ST (1998). Extractive fermentation for enhanced propionic acid production from lactose by *Propionibacterium acidipropionici*. Biotechnol Prog.

[25] Feng XH, Chen F, Xu H, Wu B, Yao J, Ying HJ, Ouyang PK (2010). Propionic acid fermentation by *Propionibacterium freudenreichii* CCTCC M207015 in a multi-point fibrous-bed bioreactor. Bioprocess Biosyst Eng.

[26] Wang Z, Jin Y, Yang ST (2015). High cell density propionic acid fermentation with an acid tolerant strain of *Propionibacterium acidipropionici*. Biotechnol Bioeng.

[27] Hsu ST, Yang ST (1991). Propionic acid fermentation of lactose by *Propionibacterium acidipropionici*: effects of pH. Biotechnol Bioeng.

[28] Stowers CC, Cox BM, Rodriguez BA (2014). Development of an industrializable fermentation process for propionic acid production. J Ind Microbiol Biotechnol.

[29] Lewis VP, Yang ST (1992). A novel extractive fermentation for propionic production from whey lactose. Biotechnol Prog.

[30] Yang ST, Huang H, Tay A, Qin W, Guzman L, San Nicolas EC, Yang ST (2007). Extractive fermentation for the production of carboxylic acids. Bioprocessing for value-added products from renewable resources.

[31] Franden MA, Pilath HM, Mohagheghi A, Pienkos PT, Zhang M (2013). Inhibition of growth of *Zymomonas mobilis* by model compounds found in lignocellulosic hydrolysates. Biotechnol Biofuels.

[32] Parizzi LP, Grassi MCB, Llerena LA, Carazzolle MF, Queiroz VL, Lunardi I, Zeidler AF, Teixeira PJPL, Mieczkowski P, Rincones J, Pereira GAG (2012). The genome sequence of *Propionibacterium acidipropionici* provides insights into its biotechnological and industrial potential. BMC Genom..

[33] Zhang A, Sun J, Wang Z, Yang ST, Zhou H (2014). Effects of carbon dioxide on cell growth and propionic acid production from glycerol and glucose by *Propionibacterium acidipropionici*. Bioresour Technol.

[34] Zou W, Zhu LW, Li HM, Tang YJ (2011). Significance of CO_2_ donor on the production of succinic acid by *Actinobacillus succinogenes* ATCC 55618. Microbial Cell Factories..

[35] Woskow SA, Glatz BA (1991). Propionic acid production by a propionic acid-tolerant strain of *Propionibacterium acidipropionici* in batch and semicontinuous fermentation. Appl Environ Microbiol.

[36] Wu Y, Shen X, Yuan Q, Yan Y (2016). Metabolic engineering strategies for co-utilization of carbon sources in microbes. Bioengineering.

[37] Ishola MM, Brandberg T, Taherzadeh MJ (2015). Simultaneous glucose and xylose utilization for improved ethanol production from lignocellulosic biomass through SSFF with encapsulated yeast. Biomass Bioenerg.

[38] Bertilsson M, Olofsson K, Lidén G (2009). Prefermentation improves xylose utilization in simultaneous saccharification and co-fermentation of pretreated spruce. Biotechnol Biofuels.

[39] Abdel-Rahman MA, Xiao Y, Tashiro Y, Wang Y, Zendo T, Sakai K, Sonomoto K (2015). Fed-batch fermentation for enhanced lactic acid production from glucose/xylose mixture without carbon catabolite repression. J Biosci Bioeng.

[40] Jiang L, Cui H, Zhu L, Hu Y, Xu X, Li S, Huang H (2015). Enhanced propionic acid production from whey lactose with immobilized *Propionibacterium acidipropionici* and the role of trehalose synthesis in acid tolerance. Green Chem.

[41] Shekiro J, Kuhn EM, Nagle NJ, Tucker MP, Elander RT, Schell DJ (2014). Characterization of pilot-scale dilute acid pretreatment performance using deacetylated corn stover. Biotechnol Biofuels.

